# Cocktail biosynthesis of triacylglycerol by rational modulation of diacylglycerol acyltransferases in industrial oleaginous *Aurantiochytrium*

**DOI:** 10.1186/s13068-021-02096-5

**Published:** 2021-12-27

**Authors:** Chuanzeng Lan, Sen Wang, Huidan Zhang, Zhuojun Wang, Weijian Wan, Huan Liu, Yang Hu, Qiu Cui, Xiaojin Song

**Affiliations:** 1 CAS Key Laboratory of Biofuels, Shandong Provincial Key Laboratory of Energy Genetics, Shandong Engineering Laboratory of Single Cell Oil, Qingdao Engineering Laboratory of Single Cell Oil, Qingdao Institute of Bioenergy and Bioprocess Technology, Chinese Academy of Sciences, No.189 Songling Road, Laoshan District, Qingdao, 266101 Shandong China; 2 Shandong Energy Institute, Qingdao, 266101 Shandong China; 3 Qingdao New Energy Shandong Laboratory, Qingdao, 266101 Shandong China; 4 Center for Ocean Mega-Science, Chinese Academy of Sciences, Qingdao, 266101 Shandong China; 5 University of Chinese Academy of Sciences, Beijing, 100049 China; 6 Faculty of Science, University of Calgary, Calgary, AB T2N 1N4 Canada

**Keywords:** *Aurantiochytrium*, Diacylglycerol acyltransferase, Polyunsaturated fatty acid, Saturated fatty acid, Thraustochytrid, Triacylglycerol

## Abstract

**Background:**

Triacylglycerol (TAG) is an important storage lipid in organisms, depending on the degree of unsaturation of fatty acid molecules attached to glycerol; it is usually used as the feedstock for nutrition or biodiesel. However, the mechanism of assembly of saturated fatty acids (SFAs) or polyunsaturated fatty acids (PUFAs) into TAGs remains unclear for industrial oleaginous microorganism.

**Results:**

Diacylglycerol acyltransferase (DGAT) is a key enzyme for TAG synthesis. Hence, ex vivo (in yeast), and in vivo functions of four DGAT2s (DGAT2A, DGAT2B, DGAT2C, and DGAT2D) in industrial oleaginous thraustochytrid *Aurantiochytrium* sp. SD116 were analyzed. Results revealed that DGAT2C was mainly responsible for connecting PUFA to the sn-3 position of TAG molecules. However, DGAT2A and DGAT2D target SFA and/or MUFA.

**Conclusions:**

There are two specific TAG assembly routes in *Aurantiochytrium*. The “saturated fatty acid (SFA) TAG lane” primarily produces SFA-TAGs mainly mediated by DGAT2D whose function is complemented by DGAT2A. And, the “polyunsaturated fatty acid (PUFA) TAG lane” primarily produces PUFA-TAGs via DGAT2C. In this study, we demonstrated the functional distribution pattern of four DGAT2s in oleaginous thraustochytrid *Aurantiochytrium*, and provided a promising target to rationally design TAG molecular with the desired characteristics.

**Supplementary Information:**

The online version contains supplementary material available at 10.1186/s13068-021-02096-5.

## Background

Oils produced by oleaginous microorganisms are an attractive lipid resource, because their manufacturing process is independence from season, climate, and location, and they can be synthesized using a wide range of carbon sources. Microbial oils include triacylglycerol (TAG), sterol esters, as well as phospholipids and glycolipids [1]. TAG is the main form of storage lipid in oleaginous cells, and each TAG molecule is formed by attaching three fatty acids to a glycerol backbone. Depending on the presence and number of double bonds in hydrocarbonated chains, fatty acids can be classified in saturated fatty acid (SFA), monounsaturated fatty acid (MUFA), and polyunsaturated fatty acids (PUFA) [2]. Thus, variety of TAG species is existed in oleaginous cells. Microorganisms and microalgae are known to be TAG producers, and some oleaginous species could synthesize more than 50% of biomass as lipids [3]. Depending on the fatty acid molecules attached to glycerol, the TAG molecules can be used for either biodiesel production or nutraceuticals [4]. TAGs in most microalgae, bacteria, and yeasts are constituted mostly by SFA and MUFA, which have been regarded as a promising alternative source for the production of biodiesel [2, 5, 6]. In addition, some of the microalgae and fungi have the ability to synthesize very long-chain PUFAs, such as eicosapentaenoic acid (EPA) and docosahexaenoic acid (DHA), which have the positive effects on human health [2, 7].

TAG is assembled from fatty acids by a class of enzymes including acyl-CoA: glycerol-sn-3-phosphate acyltransferase (GPAT), lysophosphatidate acyltransferase (LPAT), phosphatidic acid phosphatase (PAP), and diacylglycerol acyltransferase (DGAT) (Additional files 1) [8]. While DGAT is considered as a rate-limiting and key enzyme which catalyzes the final acylation of sn-1,2-diacylglycerol (DAG) to form TAG [7]. Three distinct types of DGATs referred as DGAT 1, 2, and 3 are found to take part in the biosynthesis of TAG. DGAT1 and DGAT2 are membrane-binding protein, and DGAT3 is soluble cytosolic enzyme [9]. Although they catalyze the same enzymatic reaction, DGAT1 and DGAT2 do not show any significant amino acid sequence homology with each other. In many plants, DGAT1 seems to be a more effective enzyme, whereas DGAT2 may have an important role in the formation of TAG-containing unusual fatty acids, such as PUFAs [10]. Previous study shows that overexpression of DGAT2 in *Nannochloropsis oceanica* elevates the TAG content by 69% [11]. In addition, a DGAT2 overexpression in *Chlamydomonas reinhardtii* resulted in increasing the TAG content up to ninefold [12]. Thereby, DGAT seems to represent a bottleneck in TAG biosynthesis and thus has been regarded as a key target to manipulate the TAG production. However, the mechanism regulating assembly of saturated fatty acids (SFAs) or polyunsaturated fatty acids (PUFAs) into TAGs remains unclear for industrial oleaginous microorganism. Better understanding of DGAT properties may provide new opportunities to produce the designer TAGs.

*Aurantiochytrium* is a heterotrophic marine protist whose biomass can reach to 130–150 g/L and be able to produce approximately 40%—70% (w/w) of lipids on their dry cell weight (DCW) [13–15] with the DHA content exceeds 25% of DCW [16–18]. In *Aurantiochytrium*, fatty acids are synthesized and released as free fatty acid via either fatty acid synthase or polyketide-like synthase [19], and then channeled into TAG as the storage lipid or into phospholipid as the structural lipid. The fatty acid composition of *Aurantiochytrium* is very simple under the typical fermentation condition, consisting of four major fatty acids: C14:0, C16:0, docosapentaenoic acid (DPA), and DHA [13]. Thus, lipids produced by *Aurantiochytrium* have appeared as an alternative resource to fill the gap between the demand and supply of DHA. Besides, *Aurantiochytrium* is the promising candidate for biodiesel due to its high biomass production, high lipid production, and simple lipid composition [20].

In this study, targeting the industrial oleaginous thraustochytrid *Aurantiochytrium* sp. SD116, we explored the possible functional distribution pattern of DGATs. Based on our results, we confirmed that DGAT2C was responsible for synthesis of TAG with PUFAs, and DGAT2A and DGAT2D added SFAs to TAG in *Aurantiochytrium* sp. Our study provided a promising target to rationally design TAG molecular with the desired characteristics.

## Results

### Molecular characterization of DGATs in *Aurantiochytrium*

**Fig. 1:**
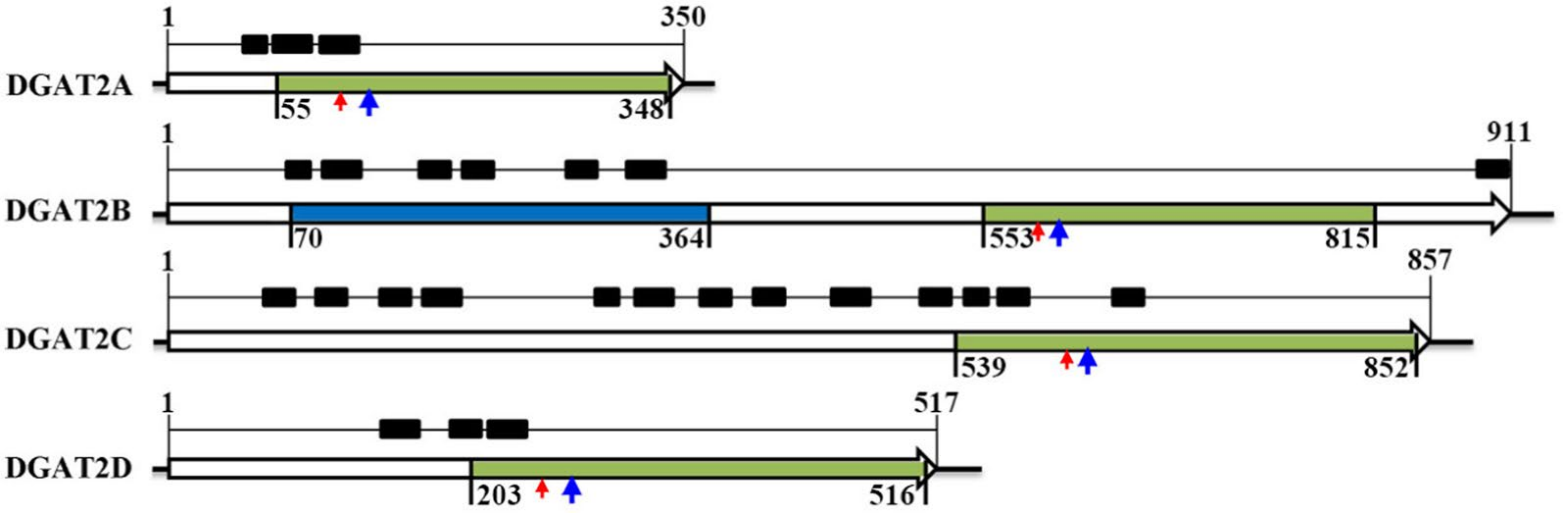
Protein structure of four DGAT2s. Black blocks stand for transmembrane regions; lines stand for amino acid sequences; green blocks represent for LPLAT superfamily domain; blue block represents for MdlB superfamily domain; red arrow indicates the “YF” motif and blue arrow indicates the “PH” motif

Four putative DGAT genes named as DGAT2A (GenBank Accession Number: MT762143), DGAT2B (GenBank Accession Number: MF926505.1), DGAT2C (GenBank Accession Number: MF926506.1), and DGAT2D (GenBank Accession Number: MF926507.1) are identified by searching the genome of *Aurantiochytrium* sp. SD116. The full length of DGAT2A, DGAT2B, DGAT2C, and DGAT2D are 350, 911,857, and 517 amino acid residues, respectively (Fig. 1). Phylogenetic analysis showed that all of these DGATs are grouped into DGAT2 family (Additional files 2). DGAT2D is tightly clustered into the subclade of animal DGAT2s, and DGAT2A is closed to *Phaeodactylum tricornutum*, while DGAT2B and DGAT2C distantly relate to other DGAT2s. Recent study has shown that DGAT2 and DGAT1 have overlapping functions in adipocytes, and the physiological role of DGAT2 in plants appears to be essential for the integration of unusual fatty acids, such as ricinoleic acid from castor and vernolic acid from ironweed into TAG. One or multiple DGAT2 genes are present in most species of microalgae, and why microalgae need many DGAT2s is still unclear. Here, four distinct ancestral origins of DGAT2s were identified in *Aurantiochytrium*, suggesting that they may have distinct functions in TAG synthesis.

Bioinformatic analysis reveal both DGAT2A and DGAT2D contain three transmembrane domains, while DGAT2B and DGAT2C contain 7 and 13 transmembrane domains, respectively (Fig. 1). All of DGAT2A, DGAT2B, DGAT2C, and DGAT2D include a lysophospholipid acyltransferase (LPLAT) superfamily domain, which is possibly involved in the acyltransferase activity (Fig. 1). DGAT2B includes another MdlB superfamily domain which is related to the ABC-type multidrug transport system, ATPase, and permease component for defense mechanisms (Fig. 1). Previous studies showed that several conserved amino acid motifs were necessary for high levels of DGAT enzymatic activity, such as the “YFP” motif and “PH” motif in the DGAT2 (encoding by *ScDGA1*) of yeast, and they were also conserved in animals and higher plants. However, in *Aurantiochytrium* sp. SD116, all four DGAT2s have the conserved “YF” motif and “PH” motif, but only DGAT2D has the complete “YFP” (Additional file 3). And both of “YF” motif and “PH” motif are located on LPLAT domain (Fig. 1).

**Fig. 2:**
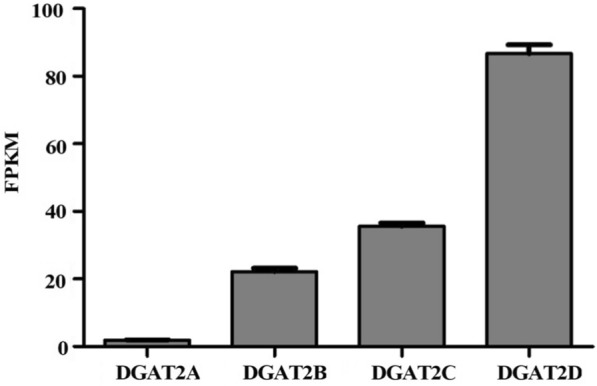
The transcriptional level of four *DGAT2s* in *Aurantiochytrium* sp. SD116

Gene transcriptional levels of DGATs in *Aurantiochytrium* sp. SD116 were observed from the transcriptome data. As shown in Fig. 2, these four genes showed variable transcriptional levels. DGAT2A and DGAT2D showed the lowest and highest transcriptional levels, respectively, and DGAT2D transcript was 47-fold higher than that of DGAT2A. DGAT2B and DGAT2C had moderate expression level, and their transcripts were 12-fold and 19-fold higher than that of DGAT2A, respectively.

### Ex vivo functional analysis of DGAT2s

**Fig. 3:**
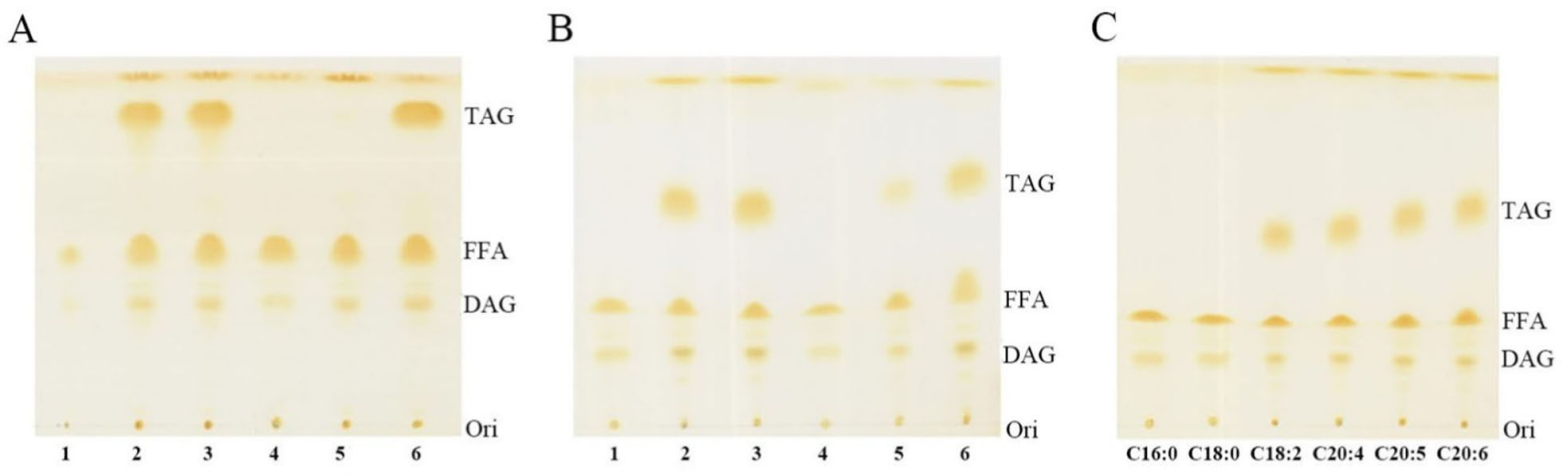
TLC analysis of lipids from H1246 and its transformants. **A** H1246 and its transformants were cultivated without fatty acid-fed. **B** H1246 and its transformants were cultivated under DHA-fed condition. Line 1, H1246 harboring the empty plasmid pYES2; line 2, H1246 expressing yeast DGA1 (DGAT2) gene; line 3, line 4, line 5, and line 6, mutant strain H1246 expressing *DGAT2A*, *DGAT2B*, *DGAT2C*, and *DGAT2D* gene, respectively. **C** TLC analysis of substrate preference of DGAT2C by feeding with C18:2, C20:4, C20:5, and C22:6

To determine the function of these four putative DGAT2 proteins, these *DGAT2s* were separately expressed in a TAG-deficient quadruple mutant stain H1246 of *S. cerevisiae* (Methods [21]), which was widely used to identify enzymes related to TAG synthesis due to its characterization of TAG-deficient [22]. The empty vector pYES2 and the yeast DGAT2 enzyme (encoding by *ScDGA1*) were used as the negative and positive controls, respectively. The correct transformants were detected by diagnose PCR (Additional file 4). As expected, TLC analysis showed that TAG was undetected in H1246 harboring the empty vector pYES2, but was appeared in transgenic H1246 cells overexpressing *ScDGA1* (Fig. 3A). A prominent TAG band was appeared in transgenic H1246 cells overexpressing *DGAT2A* or *DGAT2D*, suggesting that replenishment of these two genes could restore TAG biosynthesis in yeast mutant. In contrast, no TAG band was detected in transgenic cells expressing *DGAT2B* or *DGAT2C* (Fig. 3A). From these results, DGAT2A and DGAT2D own DGAT activity.

PUFAs, especially DHA and DPA, are the major fatty acid composition in *Aurantiochytrium* sp. SD116; however, it is absent in yeast. As shown in Additional file 5, there were four major fatty acids, including two SFAs (C16:0 and C18:0) and two MUFAs (C16:1 and C18:1) in yeast H1246. Therefore, we deduced that DGAT2B and DGAT2C did not restore TAG biosynthesis in H1246 due to the absence of appropriate substrates for TAG biosynthesis. Subsequently, DHA (C22:6) was fed to H1246 cells carrying *DGAT2s* to detect the TAG-synthetic activity. As shown in Fig. 3B, the TAG band was appeared in transgenic H1246 cells carrying *DGAT2A*, *DGAT2C*, or *DGAT2D*, while it was still undetectable in H1246 cells expressing *DGAT2B*. *DGAT2C*-carrying yeast can synthesize TAG under DHA-fed condition, suggesting that DGAT2C had the TAG-synthetic activity and it preferred the DHA as its substrate.

As shown in Fig. 1, in addition to LPLAT domain, DGAT2B has another MdlB superfamily domain which is related to the ABC-type multidrug transport system, ATPase, and permease component for defense mechanisms [23]. Therefore, we deduced that the MdlB domain may affect the TAG-synthesis activity of DGAT2B in yeast H1246. Hence, three mutants of *DGAT2B* (*mDGAT2Bs*) with deletion of MdlB domain were designed and overexpressed in yeast H1246, respectively. However, no TAG-synthetic activities were detected in *mDGAT2Bs*-carrying yeast with DHA-fed or not (Additional file 6). As we known that proper localization of protein is a key factor to exert its function. In yeast and higher plants, DGATs are located at endoplasmic reticulum (ER) for TAG synthesis [24]. Moreover, several studies report the localization of algal DGATs in chloroplast [25, 26]. Du has reported that there is no chloroplast in *Aurantiochytrium*, and DGAT2s in *Aurantiochytrium* catalyze the final step of TAG biosynthesis in ER [27]. In contrast to other DGAT2s in *Aurantiochytrium*, the LPLAT domain of DGAT2B lacks the predictable transmembrane domains that may affect the localization of mDGAT2B (Fig. 1). Considering the possible function of MdlB domain and transmembrane domains, the intact *DGAT2B* gene was overexpressed in *Aurantiochytrium* sp. SD116 to verify the functions of DGAT2B and the detailed results were displayed in the part of “*DGAT2s* overexpression in *Aurantiochytrium* sp.”.

### Detection of substrate preference of DGAT2s

**Fig. 4:**
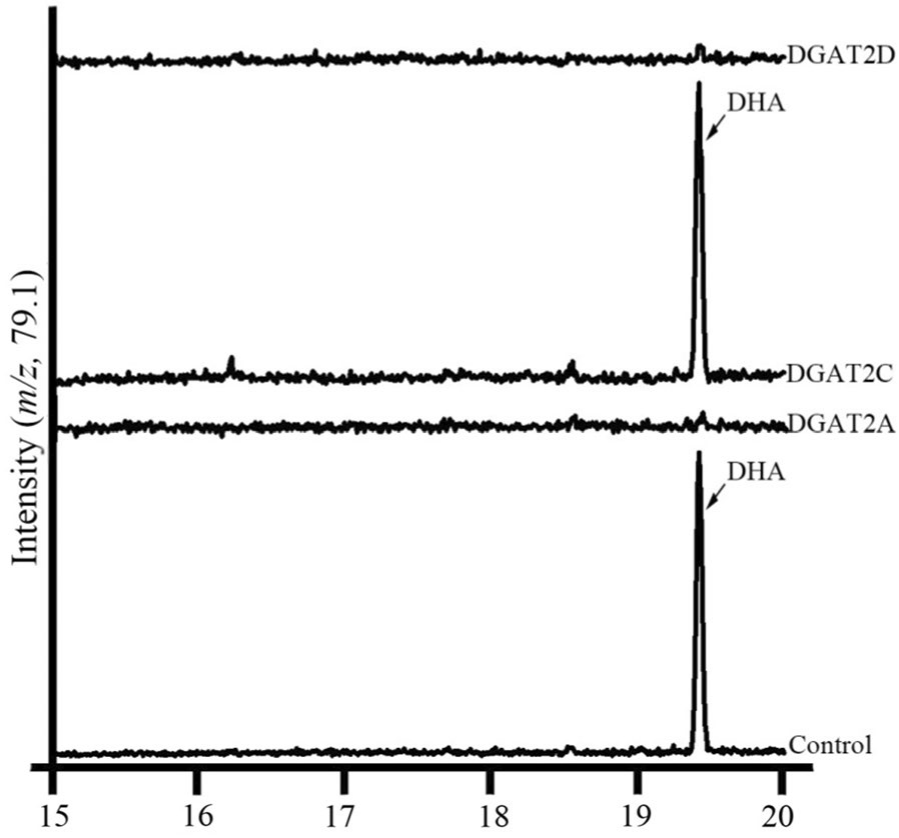
GC–MS analysis traced at m/z 79.1 of TAG products produced in the H1246 expressing *DGAT2A*, *DGAT2C*, and *DGAT2D* gene, respectively. The transformants of H1246 were cultivated under DHA-fed condition. Control, standard of methyl esters of DHA was injected

The TAG-synthetic activities of DGAT2A, DGAT2C, and DGAT2D have been confirmed. Moreover, we found that DGAT2C could target DHA instead of SFA and MUFA for assembling TAG molecule, suggesting that DGAT2C prefers DHA as its substrate. To further understand the substrate preference of DGAT2A and DGAT2D, the profiles of TAG-associated fatty acids after DHA feeding were analyzed by GC. Results showed that no DHA was appeared in the TAG products of transgenic H1246 cells carrying *DGAT2A*, and *DGAT2D*, suggesting that DGAT2A and DGAT2D prefer SFA and/or MUFA for assembling TAG (Fig. 4).

To further determine the substrate range and preference of DGAT2C, linoleic acid (LA, C18:2), arachidonic acid (ARA, C20:4), and EPA (C20:5) were also fed to H1246 cells carrying *DGAT2C*. As shown in Fig. 3C, the *DGAT2C*-carrying strain had recovered the ability to synthesize TAG in all experimental groups fed with polyunsaturated fatty acids. The *DGAT2C*-carrying yeast can synthesize TAG under PUFA-fed condition, but failed to synthesize TAG without PUFA feeding, suggesting that DGAT2C prefers the PUFAs as its substrates. In addition, DGAT2C discriminates fatty acid classes mainly based on their degree of unsaturation other than carbon chain length. However, the yeast strain expressing *DGAT2B* was still unable to recover the TAG biosynthesis when it was fed with these three PUFAs, respectively (Additional file 7).

### In vivo functional evaluation of DGAT2s

**Fig. 5:**
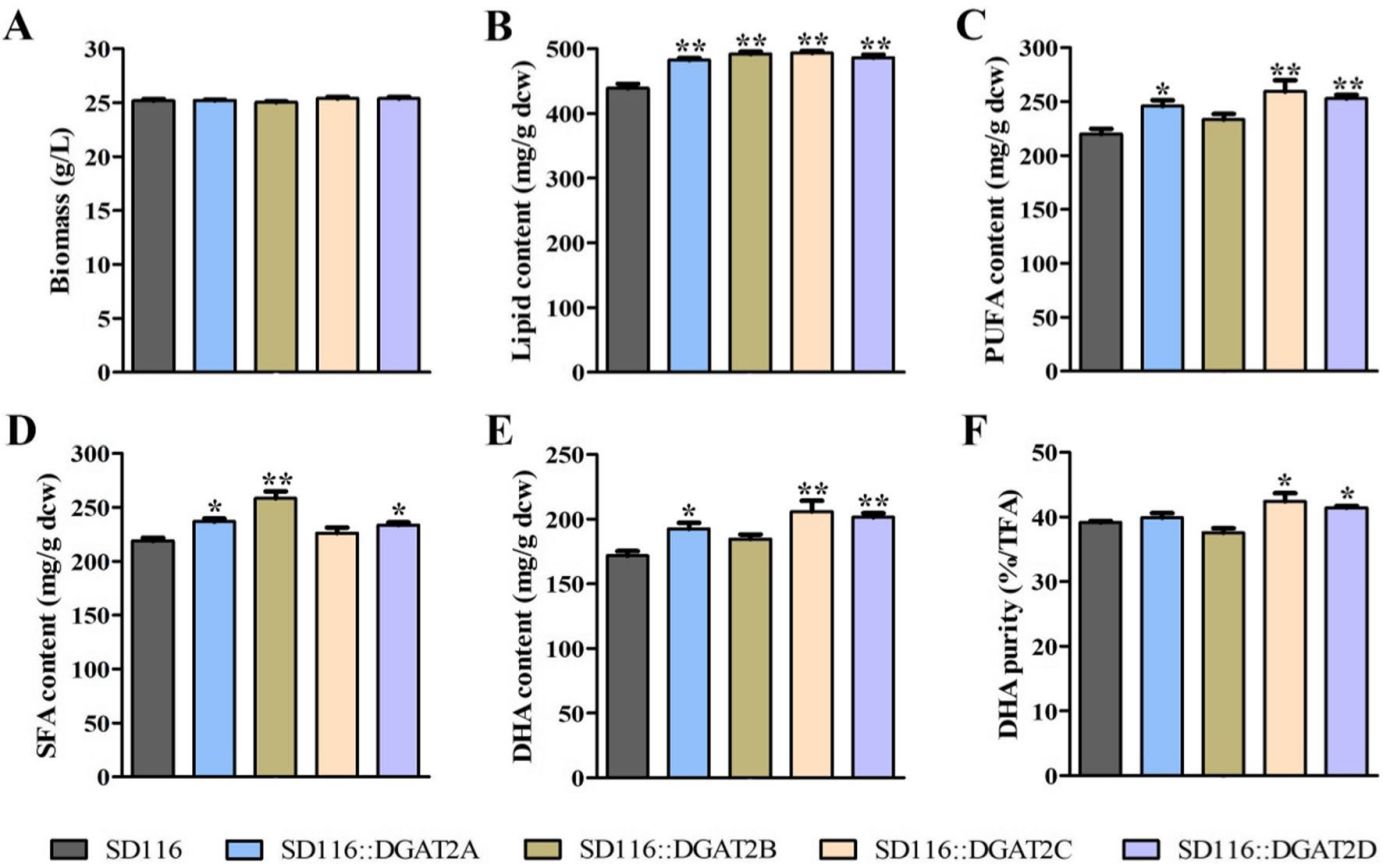
Profiles of **A** biomass, **B** lipid content, **C** PUFA content, **D** SFA content, **E** DHA content, and **F** DHA purity in SD116 and SD116::DGAT2s at stationary phase. ** P < 0.01; * P < 0.05

The in vivo function was evaluated by overexpressing these *DGAT2s* in *Aurantiochytrium* sp. SD116. As shown in Additional file 8, four *DGAT2s* were successfully expressed in *Aurantiochytrium* sp. SD116, respectively. The cell growth and glucose utilization were almost the same among SD116 and these four *DGAT2s*-overexpression mutants (SD116::DGAT2s) (Additional file 9). Although the biomass of SD116::DGAT2s was similar with that of SD116 (Fig. 5A), the lipid content in SD116::DGAT2s was significantly increased compared with that in SD116 (Fig. 5B). And the highest lipid content was observed in SD116::DGAT2C, which was 12.4% higher than that in SD116. Moreover, the lipid production in SD116::DGAT2B was also significantly improved compared with that in SD116, suggesting that DGAT2B is closely related to lipid synthesis.

Subsequently, the fatty acid profiles in SD116 and SD116::DGAT2s were analyzed. The PUFA content of SD116::DGAT2C was 17.9% higher than that of SD116 (Fig. 5C). This result suggested that overexpression of DGAT2C improved the assembly of PUFA-TAG in *Aurantiochytrium*. On the contrary, the SFA content of SD116::DGAT2B was 18.1% higher than that of SD116 (Fig. 5D). Moreover, the DHA purity in SD116::DGAT2B had a slight decrease (Fig. 5F). Based on these results, overexpression of DGAT2B appeared to improve the assembly of SFA-TAG in *Aurantiochytrium*.

Consistent with the results of ex vivo experiments in *S. cerevisiae* H1246 strain, the overexpression of *DGAT2A* and *DGAT2D *in vivo significantly promoted the synthesis of saturated fatty acids in *Aurantiochytrium*. However, overexpression of *DGAT2A* and *DGAT2D* also improved the PUFA content in *Aurantiochytrium* (Fig. 5C), and DHA purity in SD116::DGAT2D had an increase compared with that in SD116 (Fig. 5F). This phenomenon may be due to the coordinated expression regulation between DGAT genes, considering the complex and importance of lipid metabolism for *Aurantiochytrium*. Further study is needed to clarify the influence of DGAT2A and DGAT2D on TAG biosynthesis in *Aurantiochytrium,* and will provide more insights to the design of TAG moleculars.

## Discussion

Oleaginous microalgae/microorganism can be used as oil feedstocks either for biodiesel production or nutraceuticals due to their great advantages in TAG accumulation [20, 28, 29]. The physiological properties and functions of TAG molecules depend on the fatty acids composition attached to their glycerol backbone, which further determines the potential application perspective of TAG molecules. The Kennedy pathway is the main TAG-synthesis pathway, which includes four key enzymes [29]. And, DGAT is one of the most popular and promising targets for increasing TAG content in cells through metabolic engineering [30].

In contrast to most of higher plants, fungi, and animals, which encode single *DGAT2* gene, oleaginous microalgae/microbes usually contain multiple *DGAT2* genes [26]. Four putative DGAT genes were discovered through the bioinformatics analysis of the *Aurantiochytrium* genome. The phylogenetic analysis showed that four DGAT genes were distributed in three sublines, indicating that they may have different evolutionary origins (Additional file 2). DGAT2D is more closely related to animal-derived type-2 Diacylglycerol acyltransferase. This may be the reason why DGAT2D prefers to use SFA as a substrate. DGAT2A, 2B, and 2C are close to the DGATs derived from plants or microalgae. To confirm whether these *DGAT2s* are redundant copies of each other, their encoding genes were cloned and individually to detect the DGAT activity. According to the results of TLC analysis and fatty acid feeding assay in *S. cerevisiae* mutants expressing DGAT, respectively, DGAT2A, DGAT2C, and DGAT2D have the DGAT activity, while DGAT2B could not restore the TAG biosynthesis even if PUFAs were fed. By characterizing DGAT in mammals and yeasts, DGAT2 rather than DGAT1 appears to be the dominant enzyme for TAG synthesis. Chitraju et al. recently found that DGAT1 and DGAT2 have distinct and overlapping functions in adipocytes [31], while DGAT2 is not essential for TAG storage. In plant species, DGAT2 appears to be important for incorporating unusual fatty acids, such as ricinoleic acid from castor into TAG [32]. The expression of DGAT2 during embryo development was higher than that of DGAT1 from plants accumulating unusual or PUFAs. In most of the microalgal species, one or multiple DGAT2 contribute to the complexity of TAG biosynthesis. Eleven type-2 DGATs (NoDGAT2s) have been found in *Nannochloropsis oceanica* [26]. Among them, NoDGAT2A and NoDGAT2D are considered to prefer SFAs and monounsaturated fatty acids (MUFAs) as the substrates, respectively. While NoDGAT2C, 2 J and 2 K exhibited the strongest activity toward PUFAs such as linoleic acid and eicosapentaenoic acid [25, 26]. Distinct physiological functions of DGAT2s have been identified in oleaginous microalgae. Using different DGAT2s to connect different types of fatty acids to the glycerol backbone may effectively improve the efficiency of TAG synthesis.

In addition to the enzyme activity and substrate preference, the distinct expression patterns of these DGAT2s may also reflect their potential role in TAG biosynthesis. DGAT2C, which prefers the PUFAs as its substrates, has the second highest transcription level in these DGATs. DGAT2C was mainly responsible for connecting PUFA to the sn-3 position of TAG molecules. DGAT2A and DGAT2D target SFA and/or MUFA, while the transcription level of DGAT2D was much higher than those of DGAT2A. Hence, DGAT2D is considered as a major enzyme for SFA accumulation in the sn-3 position of TAG molecules.

## Conclusions

**Fig. 6:**
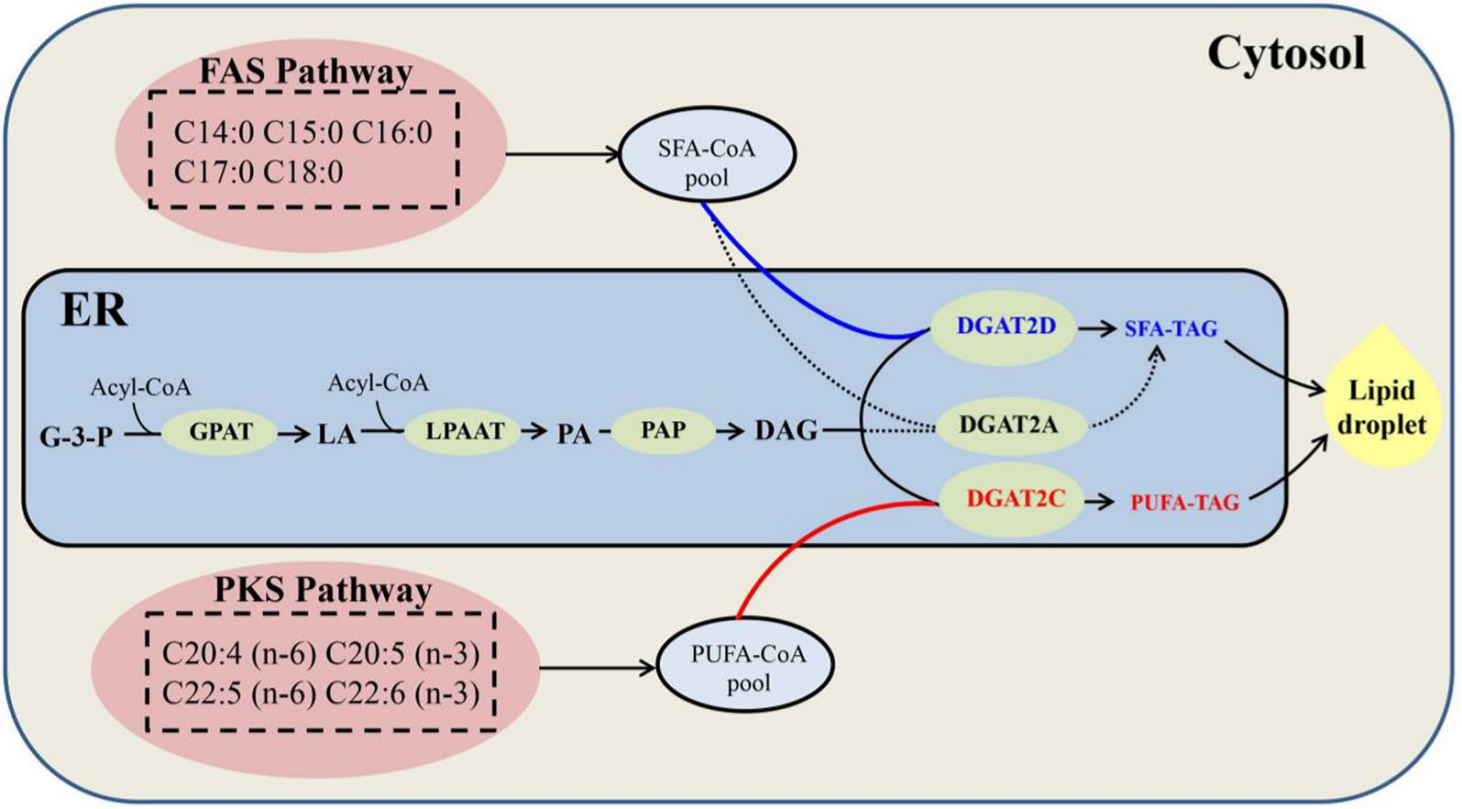
A mechanistic model of DGAT2s-mediated TAG synthesis in thraustochytrid *Aurantiochytrium* sp. *G-3-P* glycerol-3-phosphate, *LA* lysophosphatidic acid, *PA* phosphatidic acid, *DAG* diacylglycerol, *TAG* triacylglycerol, *FAS* fatty acid synthase, *PKS* polyketide-like synthase, *GPAT* glycerol-3-phosphate acyltransferase, *LPAAT* lysophosphatidic acid acyltransferase, *PAP* phosphatidic acid phosphatase [8]

Taken together, the polyphyletic origin, different but complementary substrate preference, distinct transcript abundance and product profiles, reveal the mechanism of TAG synthesis in *Aurantiochytrium* sp. There are two specific assembly routes mediated by DGAT2s for TAG synthesis in *Aurantiochytrium* sp. (Fig. 6). (1) The “SFA-TAG lane”, which prefers SFA and primarily produces SFA-TAGs mainly by DGAT2D whose function is complemented by DGAT2A. (2) The “PUFA-TAG lane”, which prefers PUFAs and primarily produces PUFA-TAGs via DGAT2C. Based on our research, recognition of each of DGATs in *Aurantiochytrium*, we could design the sn-3 position of TAG molecules and develop a “customized cell factory” for the production of microbial oils.

## Methods

### Strains and culture conditions

Strains used in this study are listed in Table 1. *Escherichia coli* DH5α was grown in Luria–Bertani (LB) medium at 37 °C with shaking at 160 rpm. *Aurantiochytrium* sp. SD116 (CGMCC no. 6208) was isolated in the previous study [13]. It was cultivated on the fermentation medium (60 g/L glucose, 20 g/L yeast extract, and 15 g/L sea salt) at 25 °C with shaking at 200 rpm. *S. cerevisiae* strain was grown on YPD medium-containing 10 g/L yeast extract, 20 g/L peptone and 20 g/L glucose, at 30 oC with shaking at 200 rpm. The *S. cerevisiae* transformants were selected on synthetic complete medium lacking uracil (SC-URA, ELITE, Shanghai, China) supplemented with 20 g/L glucose. Antibiotics is used at the following concentrations: ampicillin, 100 μg/mL; zeocin, 100 μg/mL.

**Table 1:** Strains and plasmids used in this study

Strain or plasmid	Description	Reference or source
Strains		
*Escherichia coli* DH5α	Strain used for plasmid construction	Invitrogen
*Aurantiochytrium* sp. SD116	Wild type	[13]
SD116::DGAT2A	Overexpression of DGAT2A gene in SD116	This study
SD116::DGAT2B	Overexpression of DGAT2B gene in SD116	This study
SD116::DGAT2C	Overexpression of DGAT2C gene in SD116	This study
SD116::DGAT2D	Overexpression of DGAT2D gene in SD116	This study
*Saccharomyces cerevisiae*		
H1246	The TAG-deficient quadruple of *S. cerevisiae.* Genotype: *MATα are1*– Δ::*HIS3 are2*-Δ:: *LEU2 dga1* -Δ:: *Kan MX4 lro1*-Δ::*TRP1 ADE2*	[34]
pYES2	H1246 harboring the empty plasmid pYES2	[34]
ScDGA1	H1246 expressing yeast DGA1 gene	[34]
DGAT2A	H1246 expressing DGAT2A gene	This study
DGAT2B	H1246 expressing DGAT2B gene	This study
DGAT2C	H1246 expressing DGAT2C gene	This study
DGAT2D	H1246 expressing DGAT2D gene	This study
Plasmids		
pGZC-1	Plasmid carrying the zeo^R^ gene expression cassette and 18 s rDNA homologous arm	[35]
pDGAT2A	Derived from pDGAT2C, containing DGAT2A expression cassette	This study
pDGAT2B	Derived from pDGAT2A, containing DGAT2B expression cassette	This study
pDGAT2C	Derived from pGZC-1, containing DGAT2C expression cassette	This study
pDGAT2D	Derived from pDGAT2C, containing DGAT2D expression cassette	This study

### Bioinformatic analysis

Conserved domains were performed by the CD-Search program (https://www.ncbi.nlm.nih.gov/structure/cdd/wrpsb.cgi). Predication of protein transmembrane regions was checked by the TMHMM program (http://www.cbs.dtu.dk/services/TMHMM). Phylogenetic analyses were carried out using Mega 5.0 and trees were constructed using Neighbor-Joining method.

### Plasmid construction

All primers used are listed in Additional file 10. For yeast, pYES2 vector was used to express DGATs, and it was digested with *Hind* III and *Sph* I. *DGA1* gene encoding a DGAT2 was cloned from the genome of *S. cerevisiae* with primers DGA1-F and DGA1-R, and then ligated to pYES2. *DGAT2A*, *DGAT2B*, *DGAT2C*, and *DGAT2D* were cloned from SD116 genome with primers 3465-F/3465-R, 5339-F/5339-R, 7110-F/7110-R, and 7085-F/7085-R, respectively, and then ligated into pYES2.

For *Aurantiochytrium* sp. SD116, pGZC-1 was used to express DGATs. The tubulin prompter (Ptub), *DGAT2D*, and actin terminator (Tactin) were amplified from SD116 genome with primers 1101-F/1101-R, 1102-F/1102-R, and 1103-F/1103-R, respectively. The *DGAT2D* expression cassette was constructed by overlap PCR and then ligated into the linear vector fragment which was amplified from pGZC-1 with primer pair 1104-F/1104-R to generate pDGAT2D. The construction processes of plasmids pDGAT2A, pDGAT2B, and pDGAT2C were similar to that of pDGAT2D.

### Transformation of yeasts and *Aurantiochytrium*

Expression vectors were separately transformed into yeast strain H1246 using the LiAc/SS carrier DNA/PEG method [33, 34]. Transformants were selected by growth on glucose medium (20 g/L glucose and 6.7 g/L yeast nitrogen base without amino acids) containing appropriate auxotrophic supplements.

Electrotransformation of *Aurantiochytrium* sp. SD116 was performed as previously described [35]. After electrotransformation, the cells were cultivated on solid selective medium (30 g/L glucose, 20 g/L yeast extract, 10 g/L sea salt, and 15 g/L agar) containing 50 μg/mL zeocin. The plates were incubated for 3 days in the dark at 25 °C, and the correct transformants were verified by PCR detection.

### Yeast induction and PUFA feeding

Yeast induction and PUFA feeding assay were performed as previously described [26]. Yeast was grown on glucose medium with linoleic acid (18:2), arachidonic acid (ARA), eicosapentaenoic acid (EPA), or docosahexaenoic acid (DHA). The final fatty acid concentration in medium is 90 μM with the presence of 0.1 g/L BSA. Cells were grown at 30 °C and 150 rpm for 20 h, and harvested for TAG species analysis and fatty acid composition analysis.

### Lipid isolation, TAG species analysis, and fatty acid composition analysis

Total lipids were extracted according to the previously described [35] and were finally dissolved in chloroform. Neutral lipid classes were separated from total lipids by thin-layer chromatography (TLC) in the developing agent of hexane/diethyl ether/acetic acid (80:20:1, v/v). The profiles of TAGs and DAGs from TLC plates were dissolved in chloroform and transformed to the fatty acid methyl esters (FAMEs) using the method of Cui et al. [35]. FAMEs were determined by an Agilent 7890B gas chromatograph. Separation was achieved on an HP-INNOWAX (30 m × 0.25 mm i.d., 0.25 μm film thickness), helium as the carrier gas at a constant flow rate of 1.0 mL/min. The GC temperature programming was set as our previous work. The injection and ion source temperature were both 250 °C. The mass scan range was 50–800 m/z and selected ion mode (m/z 79.1) for quantitative analysis.

## Supplementary Information

**Additional file 1: Fig. S1**. Biosynthesis pathway of triglyceride in Aurantiochytrium sp. SD116.

**Additional file 2: Fig. S2**. Phylogenetic analysis of DGATs in Aurantiochytrium sp. SD116. The phylogenetic tree was constructed according to the Neighbor-Joining (NJ) method. GenBank accession numbers are shown by following the corresponding species name.

**Additional file 3: Fig. S3**. Alignment of the conserved regions of DGAT2s. “YF” motif and “PH” motif were shown with underlined.

**Additional file 4: Fig. S4**. PCR detection. M, marker; line 1, H1246 genome; line 2, the genome of H1246 harboring the empty plasmid pYES2; line 3, the genome of H1246 expressing yeast DGA1 gene; line 4, line 5, line 6 and line 7, the genomes of mutant strain H1246 expressing DGAT2A, DGAT2B, DGAT2C and DGAT2D gene, respectively. Primers PYES2-F and PYES2-R were used for PCR detection.

**Additional file 5: Fig. S5**. Lipid profile in yeast H1246.

**Additional file 6: Fig. S6**. Analysis of lipids from H1246 and its transformants. (A) Schematic representation of the mutant strains construction. (B) TLC analysis of lipids from H1246 and its transformants that were cultivated without fatty acid-fed. (C) TLC analysis of lipids from H1246 and its transformants that were cultivated under DHA-fed condition. Line 1, H1246 expressing yeast DGA1 (DGAT2) gene; line 2, line 3, line 4 and line 5, mutant strain H1246 expressing DGAT2B, mDGAT2B-1, mDGAT2B-2 and mDGAT2B-3 gene, respectively.

**Additional file 7: Fig. S7**. TLC analysis of substrate preference of DGAT2s by feeding assay. (A) C18:2-fed; (B) ARA-fed; (C) EPA-fed. Line 1, H1246 harboring the empty plasmid pYES2; line 2, H1246 expressing yeast DGA1 (DGAT2) gene; line 3, line 4, line 5 and line 6, mutant strain H1246 expressing DGAT2A, DGAT2B, DGAT2C and DGAT2D gene, respectively.

**Additional file 8: Fig. S8**. Genomic PCR detection. M, marker; 1, SD116 genome; 2, plasmid pGZC-1; 3, SD116::DGAT2A genome; 4, SD116::DGAT2B genome; 5, SD116::DGAT2C genome; 6, SD116::DGAT2D genome. Primers Zeo-F and Zeo-R were used to verify the transformant.

**Additional file 9: Fig. S9**. Growth profile (A) and glucose utilization (B) in strains SD116 and SD116::DGAT2s.

**Additional file 10: Table S1**. Primers used in this study.

## Data Availability

All data generated or analyzed during this study are included in this published article and its Additional files 1–9.

## Abbreviations

TAG: Triacylglycerol
SFA: Saturated fatty acid
MUFA: Monounsaturated fatty acid
PUFA: Polyunsaturated fatty acid
DHA: Docosahexaenoic acid
EPA: Eicosapentaenoic acid
DPA: Docosapentaenoic acid
GPAT: Acyl-CoA: glycerol-sn-3-phosphate acyltransferase
LPAT: Lysophosphatidate acyltransferase
PAP: Phosphatidic acid phosphatase
DGAT: Diacylglycerol acyltransferase
DAG: Diacylglycerol
LB: Luria–Bertani
ARA: Arachidonic acid
TLC: Thin-layer chromatography
LPLAT: Lysophospholipid acyltransferase
LA: Linoleic acid
DCW: Dry cell weight

## References

[1] Szczepanska P, Hapeta P, Lazar Z (2021). Advances in production of high-value lipids by oleaginous yeasts. Crit Rev Biotechnol.

[2] Patel A, Karageorgou D, Rova E, Katapodis P, Rova U, Christakopoulos P, Matsakas L (2020). An overview of potential oleaginous microorganisms and their role in biodiesel and omega-3 Fatty acid-based industries. Microorganisms.

[3] Papanikolaou S (2012). Oleaginous yeasts: biochemical events related with lipid synthesis and potential biotechnological applications. Ferment Technol.

[4] Pfleger BF, Gossing M, Nielsen J (2015). Metabolic engineering strategies for microbial synthesis of oleochemicals. Metab Eng.

[5] Torres CM, Rios SD, Torras C, Salvado J, Mateo-Sanz JM, Jimenez L (2013). Microalgae-based biodiesel: a multicriteria analysis of the production process using realistic scenarios. Biores Technol.

[6] Cho HU, Park JM (2018). Biodiesel production by various oleaginous microorganisms from organic wastes. Biores Technol.

[7] Sun XM, Ren LJ, Zhao QY, Ji XJ, Huang H (2018). Enhancement of lipid accumulation in microalgae by metabolic engineering. Biochimica et biophysica acta Mol Cell Biol Lipids.

[8] Yue XH, Chen WC, Wang ZM, Liu PY, Li XY, Lin CB, Lu SH, Huang FH, Wan X (2019). Lipid distribution pattern and transcriptomic insights revealed the potential mechanism of docosahexaenoic acid traffics in *Schizochytrium* sp. A-2. J Agric Food Chem.

[9] Wagner M, Hoppe K, Czabany T, Heilmann M, Daum G, Feussner I, Fulda M (2010). Identification and characterization of an acyl-CoA:diacylglycerol acyltransferase 2 (DGAT2) gene from the microalga O. tauri. Plant Physiol Biochem PPB.

[10] Nguyen T, Xu Y, Abdel-Hameed M, Sorensen JL, Singer SD, Chen G (2019). Characterization of a type-2 diacylglycerol acyltransferase from Haematococcus pluvialis reveals possible allostery of the recombinant enzyme. Lipids.

[11] Li DW, Cen SY, Liu YH, Balamurugan S, Zheng XY, Alimujiang A, Yang WD, Liu JS, Li HY (2016). A type 2 diacylglycerol acyltransferase accelerates the triacylglycerol biosynthesis in heterokont oleaginous microalga Nannochloropsis oceanica. J Biotechnol.

[12] Hung CH, Ho MY, Kanehara K, Nakamura Y (2013). Functional study of diacylglycerol acyltransferase type 2 family in Chlamydomonas reinhardtii. FEBS Lett.

[13] Gao M, Song X, Feng Y, Li W, Cui Q (2013). Isolation and characterization of Aurantiochytrium species: high docosahexaenoic acid (DHA) production by the newly isolated microalga, Aurantiochytrium sp. SD116. J Oleo Sci.

[14] Park W-K, Moon M, Shin S-E, Cho JM, Suh WI, Chang YK, Lee B (2018). Economical DHA (Docosahexaenoic acid) production from Aurantiochytrium sp. KRS101 using orange peel extract and low cost nitrogen sources. Algal Res.

[15] Ma Z, Tan Y, Cui G, Feng Y, Cui Q, Song X (2015). Transcriptome and gene expression analysis of DHA producer Aurantiochytrium under low temperature conditions. Sci Rep.

[16] Geng L, Chen S, Sun X, Hu X, Ji X, Huang H, Ren L (2018). Fermentation performance and metabolomic analysis of an engineered high-yield PUFA-producing strain of Schizochytrium sp.. Bioprocess Biosyst Eng.

[17] Li Z, Meng T, Ling X, Li J, Zheng C, Shi Y, Chen Z, Li Z, Li Q, Lu Y (2018). Overexpression of malonyl-CoA: ACP transacylase in Schizochytrium sp. to improve polyunsaturated fatty acid production. J Agric Food Chem.

[18] Sun X-M, Xu Y-S, Huang H (2020). Thraustochytrid cell factories for producing lipid compounds. Trends Biotechnol.

[19] Metz JG, Kuner J, Rosenzweig B, Lippmeier JC, Roessler P, Zirkle R (2009). Biochemical characterization of polyunsaturated fatty acid synthesis in Schizochytrium: release of the products as free fatty acids. Plant Physiol Biochem PPB / Societe francaise de physiologie vegetale.

[20] Cui G, Wang Z, Hong W, Liu Y-J, Chen Z, Cui Q, Song X (2019). Enhancing tricarboxylate transportation-related NADPH generation to improve biodiesel production by Aurantiochytrium. Algal Res.

[21] Sandager L, Gustavsson MH, Stahl U, Dahlqvist A, Wiberg E, Banas A, Lenman M, Ronne H, Stymne S (2002). Storage lipid synthesis is non-essential in yeast. J Biol Chem.

[22] Zhang L, Zhang H, Song Y (2018). Identification and characterization of diacylglycerol acyltransferase from oleaginous fungus Mucor circinelloides. J Agric Food Chem.

[23] Prasad R, Khandelwal NK, Banerjee A (2016). Yeast ABC transporters in lipid trafficking. Fungal Genet Biol FG B.

[24] Zhang C, Iskandarov U, Klotz ET, Stevens RL, Cahoon RE, Nazarenus TJ, Pereira SL, Cahoon EB (2013). A thraustochytrid diacylglycerol acyltransferase 2 with broad substrate specificity strongly increases oleic acid content in engineered Arabidopsis thaliana seeds. J Exp Bot.

[25] Xin Y, Shen C, She Y, Chen H, Wang C, Wei L, Yoon K, Han D, Hu Q, Xu J (2019). Biosynthesis of triacylglycerol molecules with a tailored PUFA profile in industrial microalgae. Mol Plant.

[26] Xin Y, Lu Y, Lee YY, Wei L, Jia J, Wang Q, Wang D, Bai F, Hu H, Hu Q (2017). Producing designer oils in industrial microalgae by rational modulation of co-evolving type-2 diacylglycerol acyltransferases. Mol Plant.

[27] Du F, Wang YZ, Xu YS, Shi TQ, Liu WZ, Sun XM, Huang H (2021). Biotechnological production of lipid and terpenoid from thraustochytrids. Biotechnol Adv.

[28] Orozco Colonia BS (2020). Vinícius de Melo Pereira G, Soccol CR: Omega-3 microbial oils from marine thraustochytrids as a sustainable and technological solution: a review and patent landscape. Trends Food Sci Technol.

[29] Jin HH, Jiang JG (2015). Phosphatidic acid phosphatase and diacylglycerol acyltransferase: potential targets for metabolic engineering of microorganism oil. J Agric Food Chem.

[30] Chen JE, Smith AG (2012). A look at diacylglycerol acyltransferases (DGATs) in algae. J Biotechnol.

[31] Chitraju C, Walther TC, Farese RV (2019). The triglyceride synthesis enzymes DGAT1 and DGAT2 have distinct and overlapping functions in adipocytes. J Lipid Res.

[32] Kroon JT, Wei W, Simon WJ, Slabas AR (2006). Identification and functional expression of a type 2 acyl-CoA:diacylglycerol acyltransferase (DGAT2) in developing castor bean seeds which has high homology to the major triglyceride biosynthetic enzyme of fungi and animals. Phytochemistry.

[33] Gietz RD (2014). Yeast transformation by the LiAc/SS carrier DNA/PEG method. Methods Mol Biol.

[34] Liu D, Ji H, Yang Z (2020). Functional characterization of three novel genes encoding diacylglycerol acyltransferase (DGAT) from oil-rich tubers of Cyperus esculentus. Plant Cell Physiol.

[35] Cui G-Z, Ma Z, Liu Y-J, Feng Y, Sun Z, Cheng Y, Song X, Cui Q (2016). Overexpression of glucose-6-phosphate dehydrogenase enhanced the polyunsaturated fatty acid composition of Aurantiochytrium sp. SD116. Algal Res.

