# Supplementary figures and images for: Cocktail biosynthesis of triacylglycerol by rational modulation of diacylglycerol acyltransferases in industrial oleaginous Aurantiochytrium
Source: Biotechnol Biofuels. 2021 Dec 27;14:246. doi: 10.1186/s13068-021-02096-5 (PMC8714446; doi:10.1186/s13068-021-02096-5)

**
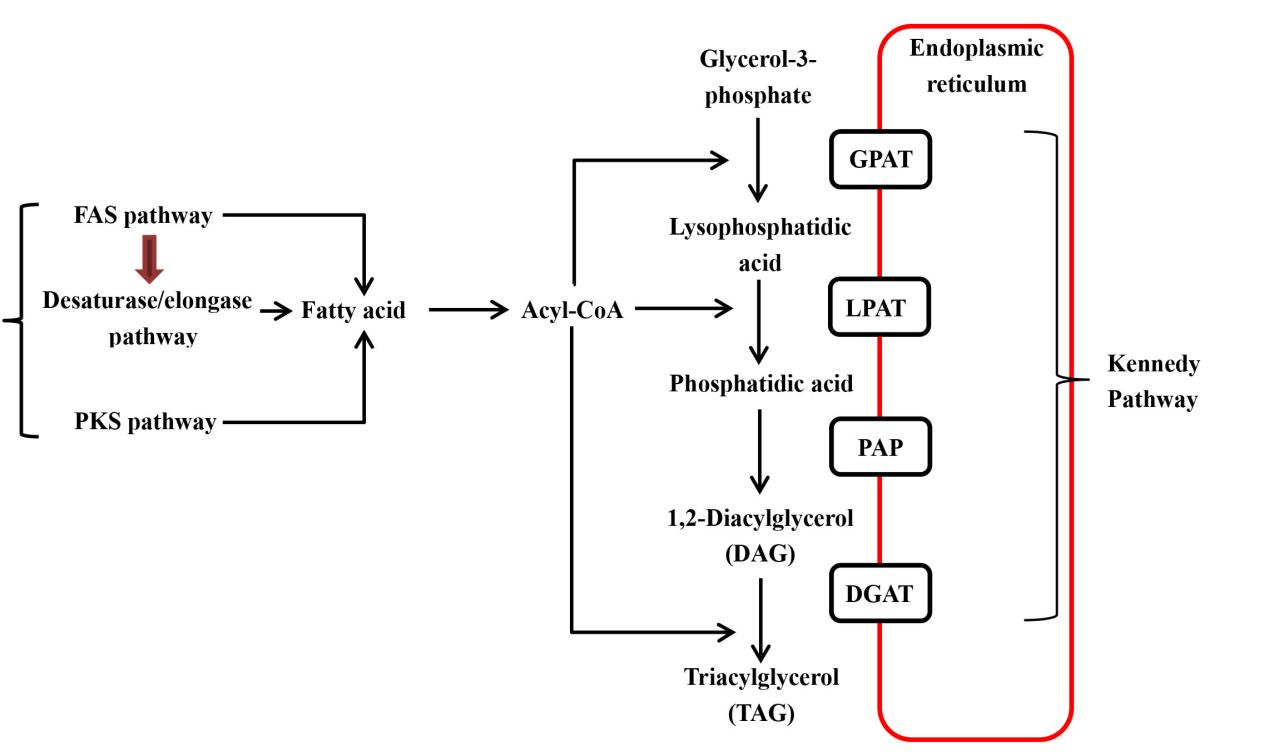
**

**Fig.S1.** Biosynthesis pathway of triglyceride in *Aurantiochytrium* sp. SD116.

Supplement: Supplementary file 1 — Additional file 1: Fig. S1. Biosynthesis pathway of triglyceride in Aurantiochytrium sp. SD116. [file 13068_2021_2096_MOESM1_ESM.docx]

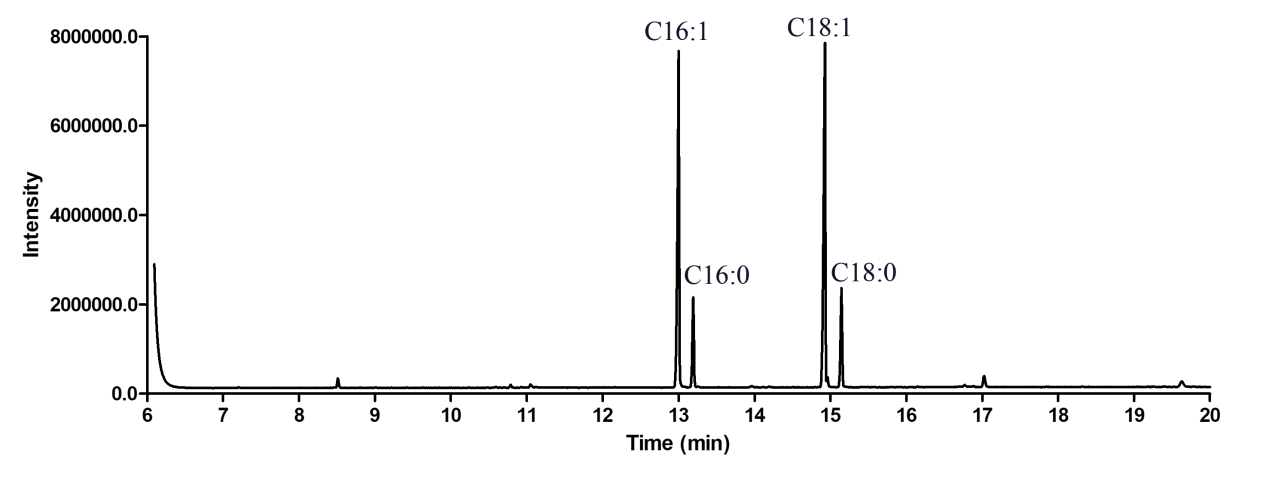


**Fig.S5.** Lipid profile in yeast H1246.

Supplement: Supplementary file 5 — Additional file 5: Fig. S5. Lipid profile in yeast H1246. [file 13068_2021_2096_MOESM5_ESM.docx]

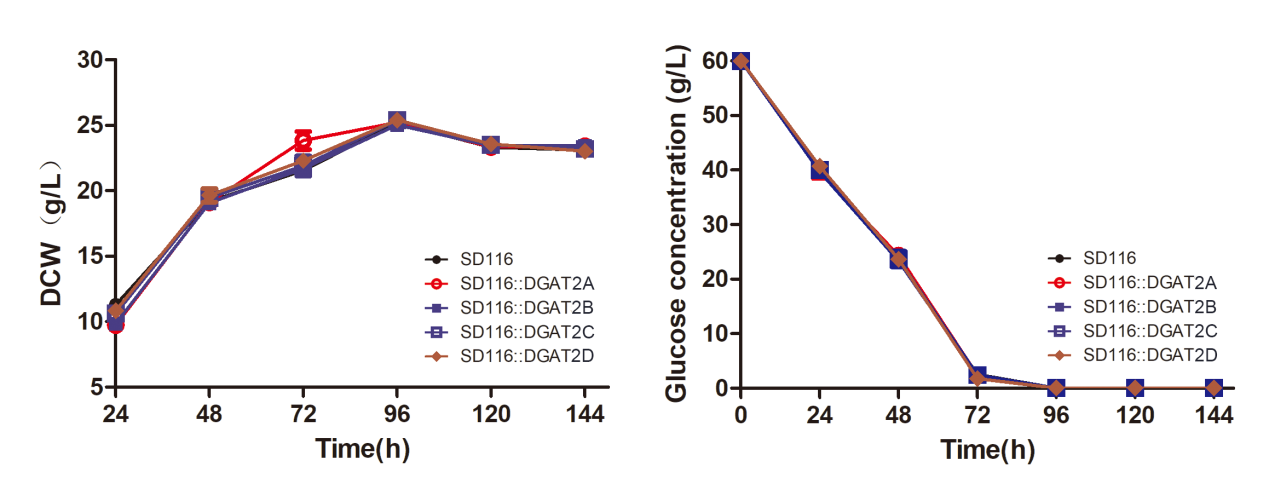


**Fig.S9.** Growth profile (A) and glucose utilization (B) in strains SD116 and SD116::DGAT2s.

Supplement: Supplementary file 9 — Additional file 9: Fig. S9. Growth profile (A) and glucose utilization (B) in strains SD116 and SD116::DGAT2s. [file 13068_2021_2096_MOESM9_ESM.docx]
