# Supplementary material for: Cocktail biosynthesis of triacylglycerol by rational modulation of diacylglycerol acyltransferases in industrial oleaginous Aurantiochytrium
Source: Biotechnol Biofuels. 2021 Dec 27;14:246. doi: 10.1186/s13068-021-02096-5 (PMC8714446; doi:10.1186/s13068-021-02096-5)
Supplement: Supplementary file 2 — Additional file 2: Fig. S2. Phylogenetic analysis of DGATs in Aurantiochytrium sp. SD116. The phylogenetic tree was constructed according to the Neighbor-Joining (NJ) method. GenBank accession numbers are shown by following the corresponding species name. [file 13068_2021_2096_MOESM2_ESM.docx]

**
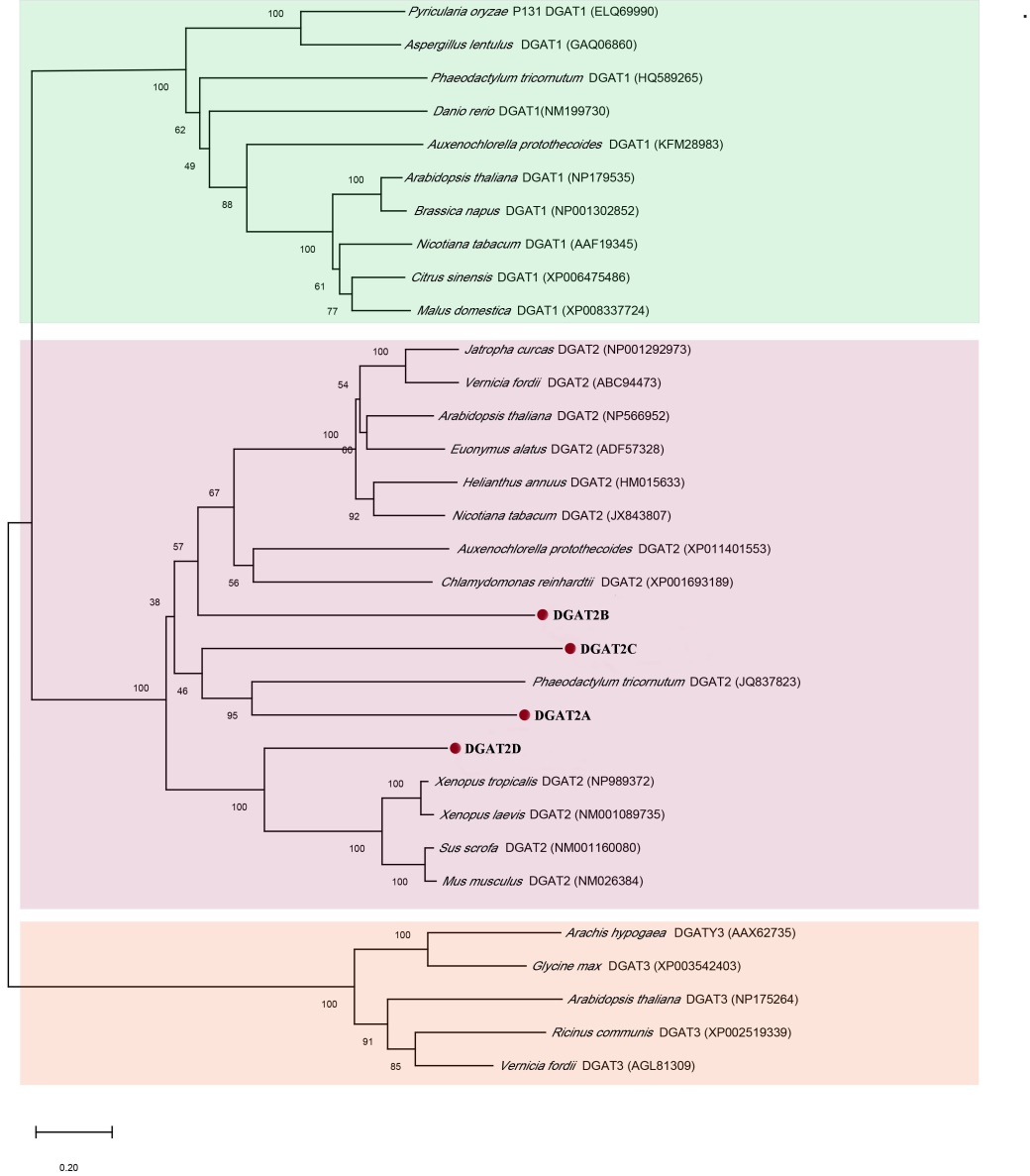
**

**Fig.S2.** Phylogenetic analysis of DGATs in *Aurantiochytrium* sp. SD116. The phylogenetic tree was constructed according to the Neighbor-Joining (NJ) method. GenBank accession numbers are shown by following the corresponding species name.
