# Supplementary material for: Cocktail biosynthesis of triacylglycerol by rational modulation of diacylglycerol acyltransferases in industrial oleaginous Aurantiochytrium
Source: Biotechnol Biofuels. 2021 Dec 27;14:246. doi: 10.1186/s13068-021-02096-5 (PMC8714446; doi:10.1186/s13068-021-02096-5)
Supplement: Supplementary file 3 — Additional file 3: Fig. S3. Alignment of the conserved regions of DGAT2s. “YF” motif and “PH” motif were shown with underlined. [file 13068_2021_2096_MOESM3_ESM.docx]

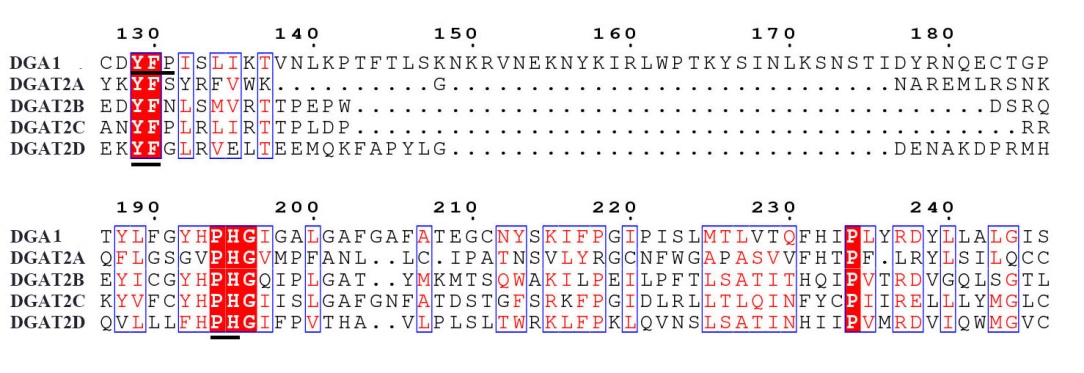


**Fig.S3.** Alignment of the conserved regions of DGAT2s. “YF” motif and “PH” motif were shown with underlined.
