# Supplementary material for: Cocktail biosynthesis of triacylglycerol by rational modulation of diacylglycerol acyltransferases in industrial oleaginous Aurantiochytrium
Source: Biotechnol Biofuels. 2021 Dec 27;14:246. doi: 10.1186/s13068-021-02096-5 (PMC8714446; doi:10.1186/s13068-021-02096-5)
Supplement: Supplementary file 4 — Additional file 4: Fig. S4. PCR detection. M, marker; line 1, H1246 genome; line 2, the genome of H1246 harboring the empty plasmid pYES2; line 3, the genome of H1246 expressing yeast DGA1 gene; line 4, line 5, line 6 and line 7, the genomes of mutant strain H1246 expressing DGAT2A, DGAT2B, DGAT2C and DGAT2D gene, respectively. Primers PYES2-F and PYES2-R were used for PCR detection. [file 13068_2021_2096_MOESM4_ESM.docx]

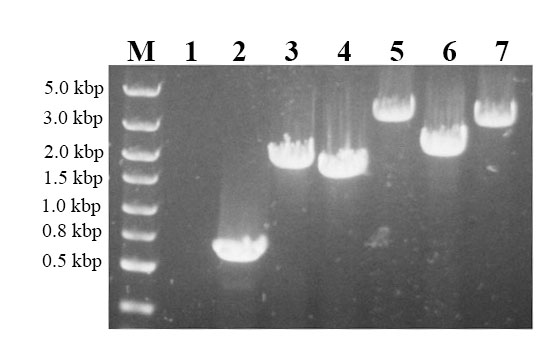


**Fig.S4.** PCR detection. M, marker; line 1, H1246 genome; line 2, the genome of H1246 harboring the empty plasmid pYES2; line 3, the genome of H1246 expressing yeast DGA1 gene; line 4, line 5, line 6 and line 7, the genomes of mutant strain H1246 expressing *DGAT2A*, *DGAT2B*, *DGAT2C* and *DGAT2D* gene, respectively. Primers PYES2-F and PYES2-R were used for PCR detection.
