# Supplementary material for: Cocktail biosynthesis of triacylglycerol by rational modulation of diacylglycerol acyltransferases in industrial oleaginous Aurantiochytrium
Source: Biotechnol Biofuels. 2021 Dec 27;14:246. doi: 10.1186/s13068-021-02096-5 (PMC8714446; doi:10.1186/s13068-021-02096-5)
Supplement: Supplementary file 6 — Additional file 6: Fig. S6. Analysis of lipids from H1246 and its transformants. (A) Schematic representation of the mutant strains construction. (B) TLC analysis of lipids from H1246 and its transformants that were cultivated without fatty acid-fed. (C) TLC analysis of lipids from H1246 and its transformants that were cultivated under DHA-fed condition. Line 1, H1246 expressing yeast DGA1 (DGAT2) gene; line 2, line 3, line 4 and line 5, mutant strain H1246 expressing DGAT2B, mDGAT2B-1, mDGAT2B-2 and mDGAT2B-3 gene, respectively. [file 13068_2021_2096_MOESM6_ESM.docx]

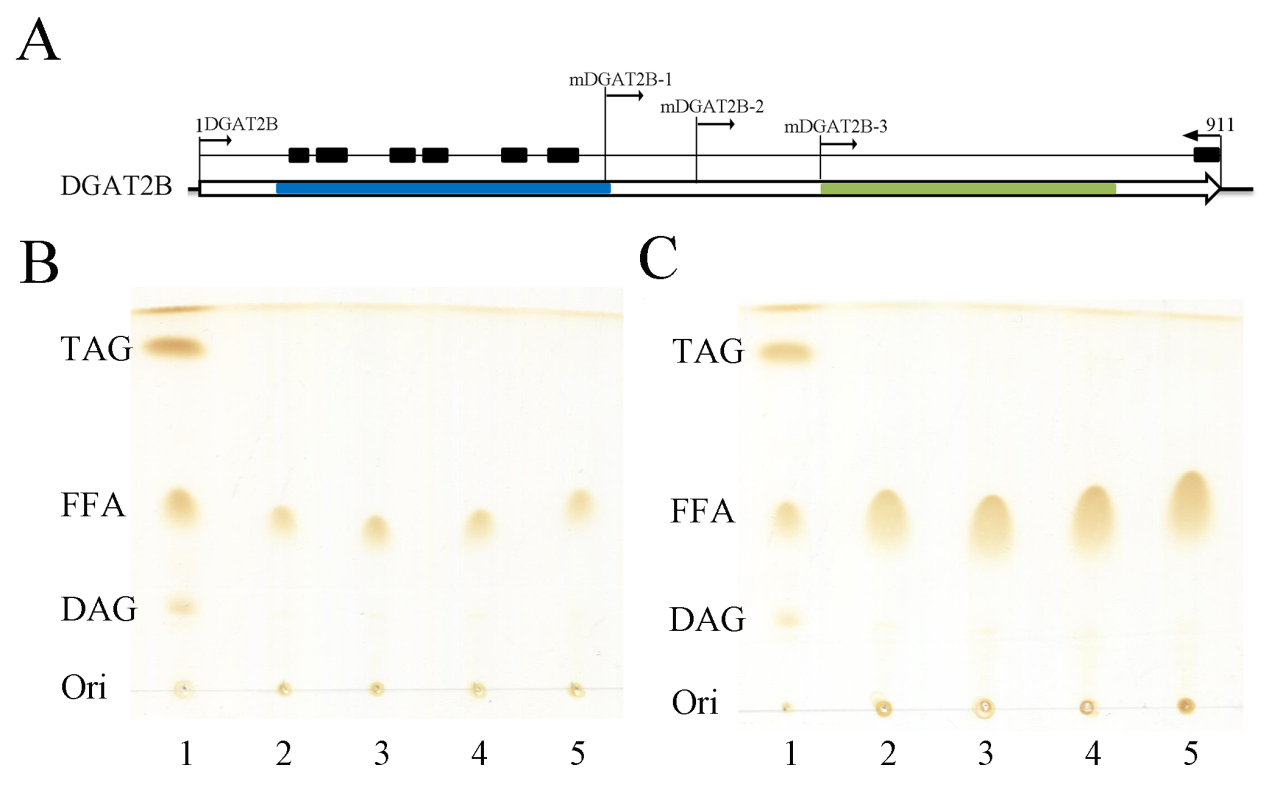


**Fig.S6.** Analysis of lipids from H1246 and its transformants. (A) Schematic representation of the mutant strains construction. (B) TLC analysis of lipids from H1246 and its transformants that were cultivated without fatty acid-fed. (C) TLC analysis of lipids from H1246 and its transformants that were cultivated under DHA-fed condition. Line 1, H1246 expressing yeast DGA1 (DGAT2) gene; line 2, line 3, line 4 and line 5, mutant strain H1246 expressing *DGAT2B*, *mDGAT2B-1*, *mDGAT2B-2* and *mDGAT2B-3* gene, respectively.
