# Supplementary material for: Cocktail biosynthesis of triacylglycerol by rational modulation of diacylglycerol acyltransferases in industrial oleaginous Aurantiochytrium
Source: Biotechnol Biofuels. 2021 Dec 27;14:246. doi: 10.1186/s13068-021-02096-5 (PMC8714446; doi:10.1186/s13068-021-02096-5)
Supplement: Supplementary file 7 — Additional file 7: Fig. S7. TLC analysis of substrate preference of DGAT2s by feeding assay. (A) C18:2-fed; (B) ARA-fed; (C) EPA-fed. Line 1, H1246 harboring the empty plasmid pYES2; line 2, H1246 expressing yeast DGA1 (DGAT2) gene; line 3, line 4, line 5 and line 6, mutant strain H1246 expressing DGAT2A, DGAT2B, DGAT2C and DGAT2D gene, respectively. [file 13068_2021_2096_MOESM7_ESM.docx]

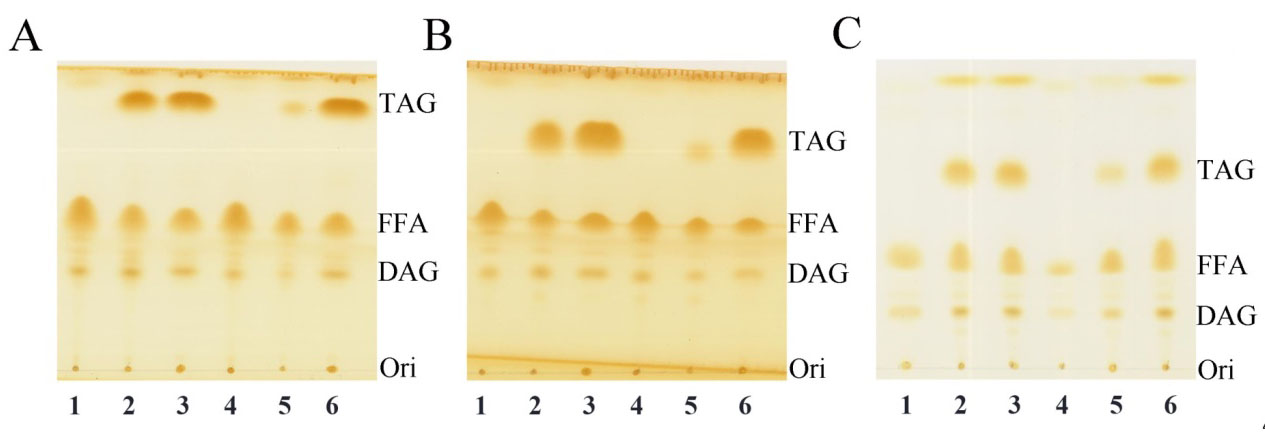


**Fig.S7.** TLC analysis of substrate preference of DGAT2s by feeding assay. (A) C18:2-fed; (B) ARA-fed; (C) EPA-fed. Line 1, H1246 harboring the empty plasmid pYES2; line 2, H1246 expressing yeast DGA1 (DGAT2) gene; line 3, line 4, line 5 and line 6, mutant strain H1246 expressing *DGAT2A*, *DGAT2B*, *DGAT2C* and *DGAT2D* gene, respectively.
