# Supplementary material for: Cocktail biosynthesis of triacylglycerol by rational modulation of diacylglycerol acyltransferases in industrial oleaginous Aurantiochytrium
Source: Biotechnol Biofuels. 2021 Dec 27;14:246. doi: 10.1186/s13068-021-02096-5 (PMC8714446; doi:10.1186/s13068-021-02096-5)
Supplement: Supplementary file 8 — Additional file 8: Fig. S8. Genomic PCR detection. M, marker; 1, SD116 genome; 2, plasmid pGZC-1; 3, SD116::DGAT2A genome; 4, SD116::DGAT2B genome; 5, SD116::DGAT2C genome; 6, SD116::DGAT2D genome. Primers Zeo-F and Zeo-R were used to verify the transformant. [file 13068_2021_2096_MOESM8_ESM.docx]

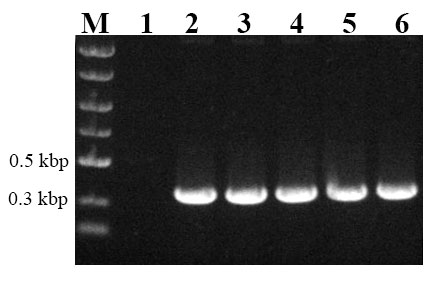


**Fig.S8.** Genomic PCR detection. M, marker; 1, SD116 genome; 2, plasmid pGZC-1; 3, SD116::DGAT2A genome; 4, SD116::DGAT2B genome; 5, SD116::DGAT2C genome; 6, SD116::DGAT2D genome. Primers Zeo-F and Zeo-R were used to verify the transformant.
