# Supplementary material for: Cocktail biosynthesis of triacylglycerol by rational modulation of diacylglycerol acyltransferases in industrial oleaginous Aurantiochytrium
Source: Biotechnol Biofuels. 2021 Dec 27;14:246. doi: 10.1186/s13068-021-02096-5 (PMC8714446; doi:10.1186/s13068-021-02096-5)
Supplement: Supplementary file 10 — Additional file 10: Table S1. Primers used in this study. [file 13068_2021_2096_MOESM10_ESM.docx]

**Table S1.** Primers used in this study.

| Primers | Sequence |
| --- | --- |
| 3465-F | agggaatattaagctcacataatggatcgcacagcagaatg |
| 3465-R | gccctatagatgcatgttacacaaagcgcaacttcc |
| 5339-F | agggaatattaagctcacataatgctgacccgcaaggttgc |
| 5339-R | gccctatagatgcatgctaaatggcgttcgaggtcg |
| 7085-F | agggaatattaagctcacataatggcggagggcgtcgaggg |
| 7085-R | gccctatagatgcatgttaatagtcgatgaagtcgag |
| 7110-F | agggaatattaagctcacataatgtcttcaagccccgctaaag |
| 7110-R | gccctatagatgcatgttacttctgaattaaaagag |
| DGA1-F | agggaatattaagctcacataatgtcaggaacattcaatgat |
| DGA1-R | gccctatagatgcatgttacccaactatcttcaattc |
| pYES2-F | ggtcgcgttcctgaaacgcag |
| pYES2-R | caagcaaggttttcagtataat |
| 1101-F | tcgaaggctttaatttgcaacaacactcacaagcaatctg |
| 1101-R | attggagtgatggaatgccct |
| 1102-F | cgtaaagcttaaacctatcaagcaaaatggcggagggcgtcgaggg |
| 1102-R | gagggcattccatcactccaatttaatagtcgatgaagtcgagctc |
| 1103-F | tttgcttgataggtttaagctttac |
| 1103-R | gcaagtcggcatcgtttacgcgtgaccttccgcgtgtcag |
| 1104-F | cgtaaacgatgccgacttgc |
| 1104-R | ttgcaaattaaagccttcga |
| 1105-F | cgtaaagcttaaacctatcaagcaaaatggatcgcacagcagaatg |
| 1105-R | gagggcattccatcactccaatttacacaaagcgcaacttccg |
| 1106-F | cgtaaagcttaaacctatcaagcaaaatgctgacccgcaaggttgc |
| 1106-R | gagggcattccatcactccaatctaaatggcgttcgaggtcg |
| 1107-F | cgtaaagcttaaacctatcaagcaaaatgtcttcaagccccgctaaag |
| 1107-R | gagggcattccatcactccaatttacttctgaattaaaagagaacctt |
| Zeo-F | atggccaagttgaccagtgcc |
| Zeo-R | tcagtcctgctcctcgg |
